# Are we all seeing the same thing? Discrepancies between parent-reported and physician-reported positional plagiocephaly severity scores

**DOI:** 10.1007/s00381-025-06833-1

**Published:** 2025-05-10

**Authors:** Grace Soojin Ryu, Mary Newland, Andrea Hiller, Elias Rizk, Thomas Samson

**Affiliations:** 1 College of Medicine, Penn State Milton S. Hershey Medical Center, Hershey, PA USA; 2 Department of Plastic Surgery, Vanderbilt University Medical Center, Nashville, TN USA; 3 Department of Neurosurgery, University of Texas at Austin, Austin, TX USA; 4 Department of Surgery, Division of Plastic Surgery, Penn State Milton S. Hershey Medical Center, Hershey, PA USA

**Keywords:** Brachycephaly, Craniofacial surgery, Pediatric, Plagiocephaly

## Abstract

**Purpose:**

Positional plagiocephaly (PP) and brachycephaly are conditions characterized by head flattening. There has been a sharp rise in the number of patients diagnosed since the American Academy of Pediatrics initiated the “Back to Sleep” policy to combat sudden infant death syndrome. This study compares providers’ and guardians’ perceived head shape differences, highlighting how these scores can alleviate parental anxiety.

**Methods:**

A retrospective chart review was performed for all pediatric patients seen for a PP consult from January 2018 to November 2023. Fifty-nine patients (43 with plagiocephaly and 16 with brachycephaly) met the inclusion criteria, in which documentation recorded two severity scores, one rating each by the provider and parental guardian. Patient demographics, severity scores, and comorbidities were recorded. The institution utilized validated, qualitative assessment forms that evaluated plagiocephaly on a 15-point scale and brachycephaly on a 9-point scale.

**Results:**

For plagiocephaly, the providers and guardians rated severity with a median of 4 (IQR 3–4.5) and 4 (IQR 3–7), respectively (Wilcoxon signed rank test, *p*-value < 0.05). For brachycephaly, the providers and guardians rated severity with a mean of 3.59 (SD 1.28) and 4.69 (SD 1.66), respectively (paired *T*-test, *p*-value < 0.005).

**Conclusions:**

Our study highlights the similarities in scores assessing clinical severity between providers and parents evaluated in a standardized, qualitative assessment for PP. On average, plagiocephaly reflected a “mild” severity, while brachycephaly reflected a “mild” to “moderate” severity on a graded scale. Future studies are needed to determine how patient-provider interactions may influence parents’ scores through shared decision-making.

## Introduction

Head shape abnormalities are one of the most common concerns pediatric clinicians will encounter [1]. While most head shape abnormalities are benign and will resolve independently, some are more serious and require surgical correction. The latter is a condition called craniosynostosis and is due to a pathological premature fusion of sutures, which restricts brain growth and requires surgical treatment. A benign condition that presents similarly to craniosynostosis is plagiocephaly, which broadly refers to head asymmetry [2]. Non-synostotic, positional plagiocephaly (PP) is characterized by unilateral occipital flattening and cranial asymmetry. A variant of this is brachycephaly, which is bilateral occipital flattening, causing a wide head shape [3]. Plagiocephaly is typically due to mechanical forces, such as positioning during sleep or feeding, that can mold the infant’s skull and cause a misshapen head. This generally resolves with positioning corrections; in some cases, helmeting may be necessary [4].

There has been a sharp rise in this condition, with an estimated 48% of infants in the USA affected since the American Academy of Pediatrics initiated the “Back to Sleep” policy to combat sudden infant death syndrome (SIDS). This initiative encouraged parents to position infants in the supine position for sleep. As a result, the incidence of SIDS decreased by 40%, but the incidence of PP increased by approximately 600% [4, 5]. Physicians have reported seeing more parents presenting with concerns about head shape abnormalities in their infants. Children with head shape abnormalities require timely referral to a pediatric neurosurgeon or plastic surgeon to rule out the diagnosis of craniosynostosis, which requires prompt surgical management. While head shape abnormalities are a routine and non-concerning consult to many physicians, it is not routine for the new parent. Abnormal head shape causes stress on the parents or guardians, prompting evaluation [5].

Children with positional plagiocephaly detected before 2 months of age with congenital muscular torticollis can benefit from early physical therapy for torticollis. All children with torticollis have been encouraged to engage in stretching exercises if they have not sought counseling from the patient’s pediatrician [4]. Previous studies have also supported the supplementary benefit of helmet therapy if clinical severity of PP was “severe” or in conjunction with surgical treatment for craniosynostosis, which is not associated with positional plagiocephaly [4]. Helmet therapy for moderate to severe positional plagiocephaly has been previously recommended if discovered at a later stage or if concerns persist after physical therapy [6]. Additionally, physical therapy and manual therapy have been recommended as the first line for clinical severity beyond mild, such as techniques involving mobilization of cranial structures optimizing range of motion and length [7].

Positional plagiocephaly and associated parental anxiety surrounding their child’s abnormal head shape can be prevented with proper patient and family education. Early evaluation and diagnosis have been proven to reduce the emotional burden and financial strain. One study by Watt et al. hypothesized that diagnosis at 4 months of age results in a treatment cost of $1495, while diagnosis only 2 months later results in a cost of $5195 [8]. These costs include an average of $1200 per family for physical therapy, $1500–$3000 for cranial orthoses, and clinical appointments or follow-up visits that may have variable insurance coverage. While this discrepancy is unclear, Lam et al. [9] discuss that insurance coverage has a significant influence. The coverage for low-income Americans often does not support treatment plans requiring multiple visits, which impairs accessibility and can lead to the development of more severe cranial deformities.

Patients on Medicaid were 1.30 times more likely to have a delayed presentation for helmet therapy than those with commercial insurance. In contrast, patients in higher income brackets were 1.45 to 1.55 times more likely to receive helmet therapy following consultation [10]. This supports the fact that there are differences in care between different socioeconomic classes. Therefore, an effort should be made to expedite early diagnosis and parent education to reduce emotional burden and financial costs.

Furthermore, the efficacy of orthotic devices has been limited, with weak evidence of a short-term benefit that are similar to improvements reported with conservative therapies such as repositioning techniques [11–13]. A parent may seek guidance on how to navigate a PP diagnosis with concerns including how various treatments, including helmets, headbanding, and conservative observation and repositioning strategies, may change the overall cosmetic appearance of their child’s head and possible teasing and psychological burden their child may experience due to their peers’ comments [11]. In a study where parents assessed the cosmetic deformity of head shape’s when they were at least 5 years of age, there were no significant differences between children with or without helmet or headband use [11]. Additionally, 58% of parents reported residual asymmetry but less than half of these parents reported concerns regarding appearance [11].

Cranial asymmetry, particularly non-synostotic plagiocephaly and brachycephaly, is a common concern among parents and often leads to consultation with craniofacial specialists. While the clinical significance and management strategies for these conditions have been widely discussed, less is known about the differences in perception between guardians and providers regarding the severity of deformity. These perceptual differences may contribute to heightened parental anxiety and influence treatment preferences. A better understanding of these differences can help optimize counseling and improve the quality of shared decision-making. The primary aim of this study is to directly compare providers’ and guardians’ perceived head shape differences using severity scores for both non-synostotic plagiocephaly and brachycephaly. A secondary aim is to assess how the degree of alignment or discrepancy in these perceptions can be used to support shared decision-making, guide patient counseling, and provide reassurance to caretakers.

## Methods

This pilot study was conducted by a neurosurgeon and craniofacial plastic surgeon participating in a biweekly craniofacial clinic at a single institution to explore associations between provider and parental severity scores in plagiocephaly and brachycephaly. Two of the three attendings, two plastic surgeons and one neurosurgeon, participating in the clinic evaluated all patients. At each consultation, guardians were asked to fill out one of two forms evaluating the degree of asymmetry for the diagnosis of concern and providers independently completed the survey at the end of the visit. Forms were then scanned into the patients’ chart.

**Fig. 1:**
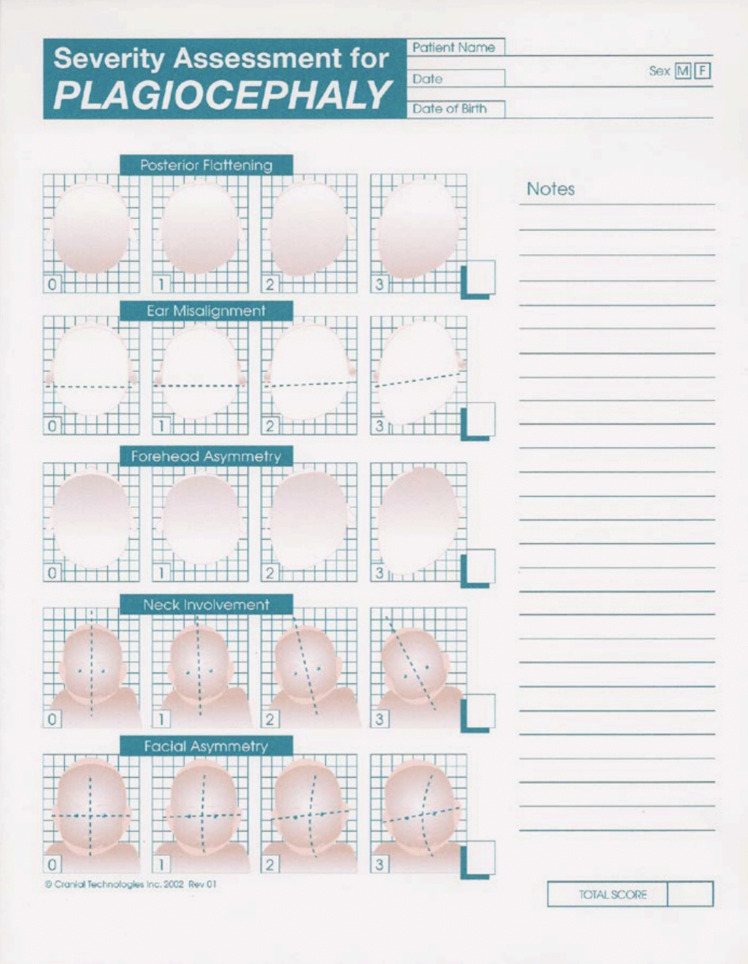
The severity assessment for plagiocephaly used at institution

**Fig. 2:**
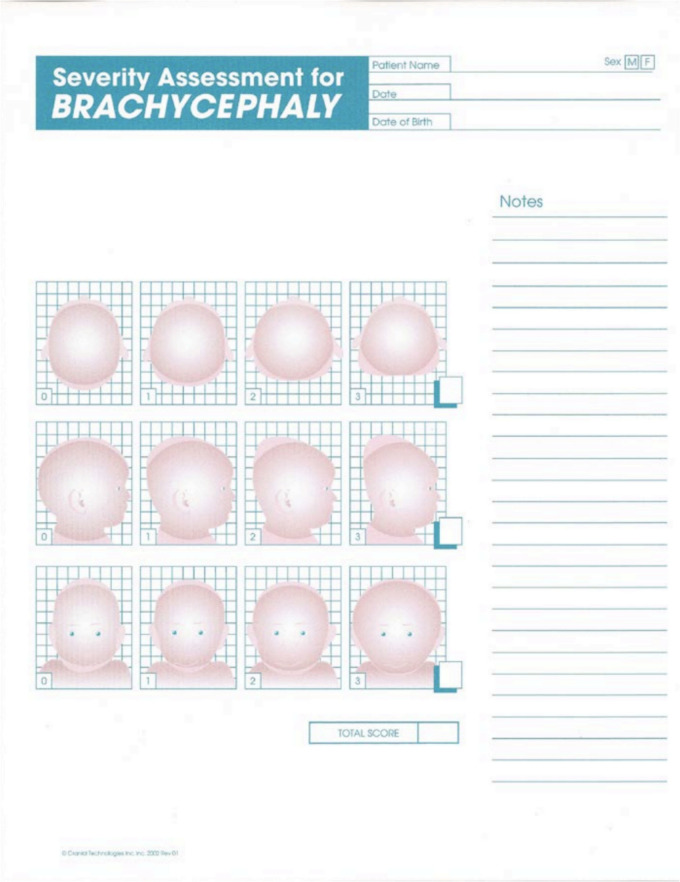
The severity assessment for brachycephaly used at institution

The Severity Assessment for Plagiocephaly and Brachycephaly developed by the Cranial Technologies Assessment form was utilized as a qualitative measure for both providers and parents to assess the degree of asymmetry depicted by a series of pictures, corresponding to increased degree of asymmetry (Figs. 1 and 2) [14].

In plagiocephaly, there were five domains assessed: posterior flattening, ear misalignment, forehead asymmetry, neck involvement, and facial asymmetry. Each domain was scored from 0 to 3, 0 representing the absence of the characteristic and 3 representing the most severe degree of asymmetry. Scores ranged from 0 to 15 for plagiocephaly [8]. The clinical grade is as follows: 0–5, 6–10, and > 10 for mild, moderate, and severe clinical categories of plagiocephaly.

Brachycephaly was evaluated on a 9-point scale. There were three domains assessed for brachycephaly: plagiocephaly posterior flattening, brachycephaly posterior flattening, and brachycephaly lateral view flattening. Similar to the grading system for plagiocephaly, each domain was scored from 0 to 3. The clinical grade is as follows: 1–3, 4–6, and 7–9 for mild, moderate, and severe clinical categories of brachycephaly.

A retrospective chart review included all pediatric patients seen for a positional, non-synostotic plagiocephaly or brachycephaly consult or concerns about an “abnormal head shape” from January 2018 to November 2023. For both the plagiocephaly and brachycephaly cohorts, the patient’s sex, race, and age at initial consult were collected (Tables 1 and 2, respectively). The mean provider and guardian severity scores (and scores by domain) were collected. Both non-parametric and parametric tests were performed according to the distribution of data, and the weighted Kappa correlation was calculated (Tables 3 and 4). Patients’ data were excluded if one of two forms were missing by the provider or guardian.

**Table 1:** Demographics for plagiocephaly cohort

Plagiocephaly demographics		
	Number of patients	Percentage
Male	28	65.10%
Female	15	34.90%
Average age (months)	6.023	
Race		
White	26	60.50%
Unknown	14	32.60%
Hispanic or Latino	1	2.33%
Asian	0	0.00%
Other	2	4.65%

**Table 2:** Demographics for brachycephaly cohort

Brachycephaly demographics		
	Number of patients	Percentage
Male	13	81.20%
Female	3	18.80%
Average age (months)	6.19	
Race		
White	6	37.50%
Unknown	6	37.50%
Hispanic or Latino	0	0.00%
Asian	4	25.00%

**Table 3:** Severity scores and breakdown of scores for plagiocephaly cohort

Plagiocephaly severity scores			
	Provider	Guardian	*p*-value
Mean severity score ranking	3.81	4.95	0.01461
Mean posterior flattening score	1.46	1.52	0.59246
Mean ear misalignment score	1.31	1.21	0.40387
Mean forehead asymmetry score	0.536	0.925	0.00362
Mean neck involvement score	0.345	0.884	0.0011
Mean facial asymmetry score	0.214	0.564	0.00461
Weighted Kappa correlation (CI)	0.455 (0.256–0.65)

**Table 4:** Severity scores and breakdown of scores for brachycephaly cohort

Brachycephaly severity scores			
	Provider	Guardian	*p-*value
Mean severity score ranking	3.59	4.69	0.0044
Mean plagiocephaly posterior flattening score	1.63	1.77	0.30075
Mean brachycephaly posterior flattening score	1.20	1.77	0.001
Mean brachycephaly lateral view flattening score	0.8	1.13	0.09614
Weighted Kappa correlation (CI)	0.454 (0.076–0.83)		

## Results

### Demographics and study characteristics

**Fig. 3:**
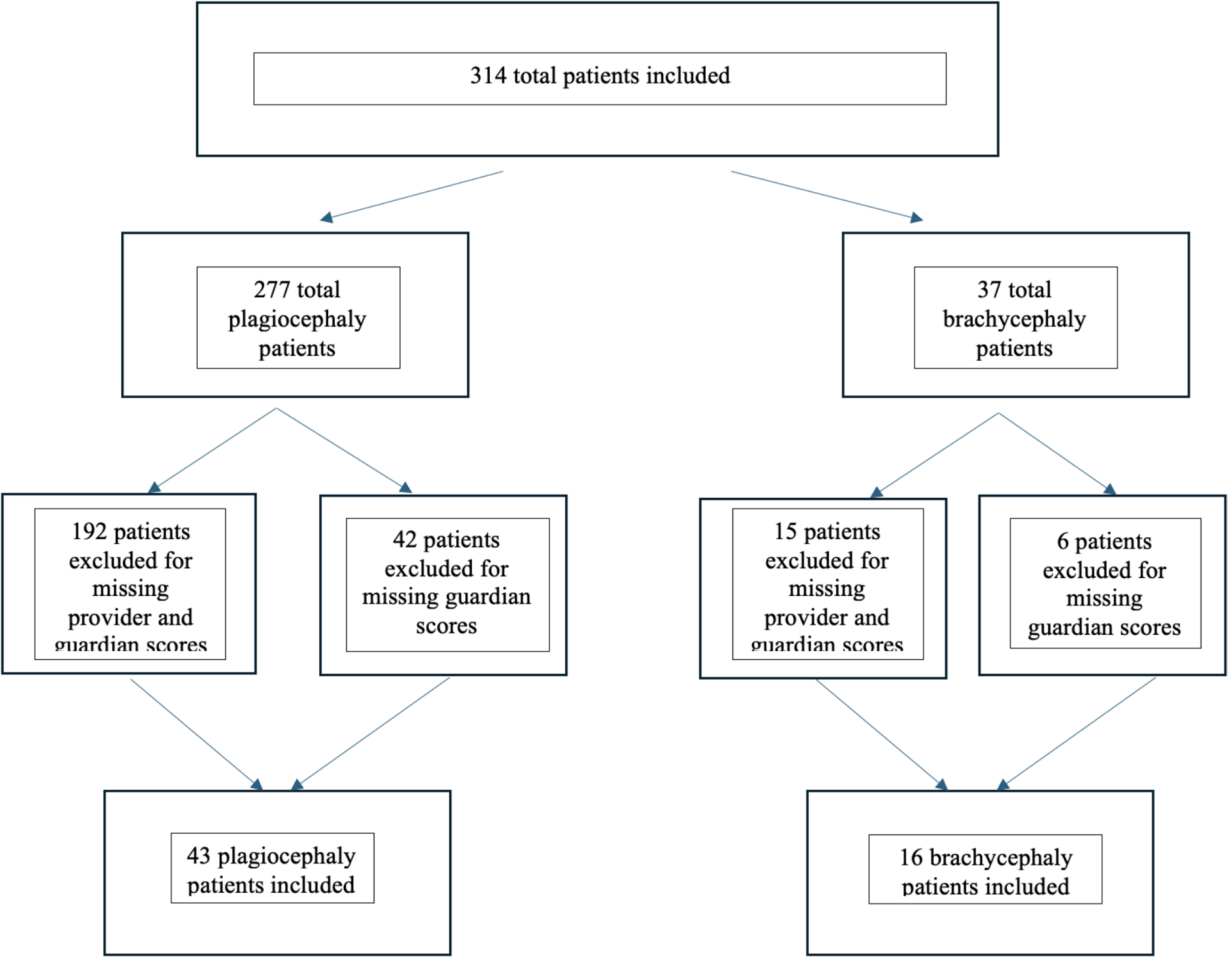
Inclusion and exclusion criteria of selected plagiocephaly patients

During the study period, three hundred fourteen patients were seen in consultation for abnormal head shape. Fifty-nine patients, 43 for plagiocephaly and 16 for brachycephaly, met the inclusion criteria and were included in the final analyses. Patients were excluded if the severity scores were not recorded, by either provider or guardian, or scanned into the electronic health records. A reflection of this exclusion process is detailed in Fig. 3. The cohort consisted of 18 female patients (3 females with brachycephaly and 15 females with plagiocephaly) and 41 male patients (13 female patients with brachycephaly and 28 females with plagiocephaly). There were 16 patients and 2 patients with torticollis for plagiocephaly and brachycephaly, respectively. The demographics are outlined in Tables 1 and 2.

### Severity score analysis

For patients with plagiocephaly, the providers and guardians rated severity with a median of 4 (IQR 3–4.5) and 4 (IQR 3–7), respectively (Wilcoxon’s signed rank test, *p*-value < 0.05). The weighted Kappa correlation coefficient was 0.455 (0.256–0.65). For brachycephaly, the providers and guardians rated severity with a mean of 3.59 (SD 1.28) and 4.69 (SD 1.66), respectively (paired *T*-test, *p*-value < 0.005). The weighted Kappa correlation coefficient was 0.454 (0.076–0.83). A Wilcoxon signed rank test was selected for analysis for patients with plagiocephaly because of the non-normal distribution of severity scores. For patients with brachycephaly, a paired *T*-test was selected because of the normal distribution of severity scores. These results are outlined in Tables 3 and 4.

## Discussion

Though statistical differences in the numerical values of the severity scores were apparent for both conditions, outcomes measured at this institution reveal that guardian and clinician observations are quite similar in the clinical context. For plagiocephaly, providers and guardians evaluated severity as mild, whereas for brachycephaly, severity was rated between mild and moderate by providers and in the moderate category by parents. While these differences in mean scores reached statistical significance, they remained within similar clinical category ranges, suggesting limited impact on treatment decisions, particularly since conservative management is the standard of care for these severity levels. Nevertheless, understanding this discrepancy can be important when counseling families, as parents perceiving higher severity may be more anxious or inclined to seek intervention. Our study exemplifies similarities in qualitative observations between guardians and providers, reinforcing the opportunity for shared understanding during initial consultations.

Since observational management has remained the first line of treatment at this institution for mild to moderate severity, guardians are offered follow-up visits if initial concerns persist. Interestingly, no patients in this cohort who met the inclusion criteria returned for a second appointment. This could indicate that initial counseling effectively addressed parental concerns and minimized the perceived need for further visits; however, we cannot rule out the possibility of patients being lost to follow-up, possibly seeking care elsewhere that may offer different treatment modalities. Because our study’s inclusion criteria required two assessments by both providers and parents with scoring domains complete for each of the domains of plagiocephaly and brachycephaly, our study cohort size was small, and patients in this study cohort may not be representative of patients that may return for follow-up visits.

Severity scores by providers did not exceed the “moderate” category of clinical severity, so helmet therapy was not recommended, which is consistent with recommendations from a recent randomized controlled trial [13]. Moreover, the American Academy of Pediatrics (AAP) guidelines suggest that helmet therapy is preferred if preventative positioning measurements are ineffective. Helmet therapy should be started before 12 months of age, and other causes of abnormal head shape should be ruled out with an ultrasound before treatment [15].

During the initial consultation with the surgeons, parents may express concerns about their child’s developing head shape. Our study shows that, numerically, parents evaluate a greater degree of asymmetry than providers, and with the wide range of therapies available, these visits may heighten parental anxiety. Although severity scores differed—with parents assigning higher scores—our findings also suggest that parents have a relatively accurate perception of severity, which can be harnessed to foster empathetic dialogue during clinical consultations. This emphasizes the importance of providers validating and acknowledging mild to moderate asymmetry, as these shared perceptions can be used to guide families through evidence-based recommendations. Specifically, clinicians can use these insights to reinforce the efficacy of conservative management approaches, especially when parents are anxious about the appearance or severity of head shape abnormalities.

Clinical applications include but are not limited to providers implementing structured communication tools or visual aids that align parent and provider assessments, creating a shared understanding early in the decision-making process. Parental concerns should be directly addressed with intentional, clear conversations that reduce the perceived need for unnecessary interventions and alleviate the financial burden of treatment. Additionally, offering follow-up visits at the parents’ discretion can help monitor progress and further reduce parental anxiety, creating a more supportive and informed clinical environment.

During these follow-up visits, future studies might explore whether score differences between parents and providers diminish over time as parental understanding improves. Further research should also investigate the development and effectiveness of interventions—such as decision aids, digital scoring tools, or caregiver education modules—that aim to harmonize expectations between providers and parents. These efforts may enhance shared decision-making, reduce unnecessary treatment, and ultimately improve patient and family satisfaction in craniofacial care.

## Conclusions

Our study highlights the similarities in scores assessing clinical severity between providers and parents evaluated in a standardized, qualitative assessment for PP. On average, plagiocephaly reflected a “mild” severity, while brachycephaly reflected a “mild” to “moderate” severity on a graded scale. Future studies are needed to determine how patient-provider interactions may influence parents’ scores through shared decision-making.

## Data Availability

No datasets were generated or analysed during the current study.
